# Supplementary material for: Invasive and Non-Invasive Neuromodulation for the Treatment of Substance Use Disorders: A Review of Reviews
Source: Brain Sci. 2025 Jul 6;15(7):723. doi: 10.3390/brainsci15070723 (PMC12294003; doi:10.3390/brainsci15070723)
Supplement: Supplementary file 1 [file brainsci-15-00723-s001.zip › brainsci-3676613-supplementary.pdf]

Invasive and Non-Invasive Neuromodulation for the Treatment of Substance Use Disorders: A Review of Reviews

SUPPLEMENTARY MATERIALS

SEARCH STRATEGY

Development

The search strategy was developed in Embase (Ovid) by a medical librarian (MM).

Limits and filters

English language database limits were applied as available or built into searches when possible. An unpublished filter was used to limit study design to systematic review with or without meta-analysis. Conference abstracts and conference reviews were excluded via command line search. Subject areas and categories were utilized as limits in Scopus and Web of Science databases.

Peer Review

The Embase search query was reviewed by an independent medical librarian.

DATABASES, SEARCH ENGINES, AND TRIAL REGISTER SEARCHES

Embase 1974 to 2024 December 19 (Wolters Kluwer Ovid interface)

Date searched: December 20, 2024

Records retrieved: 347

Language: limited to English

Study design: limited to systematic review with or without meta-analysis

Publication type: excluded conference abstract and conference review

(((alcoholism/ OR amphetamine dependence/ OR benzodiazepine dependence/ OR cocaine dependence/ OR drug dependence/ OR methamphetamine dependence/ OR exp narcotic dependence/ OR phencyclidine dependence/ OR alcoholism.ti,ab,kf. OR (addict OR addicts OR alcoholic*).ti,kf OR ((alcohol or amphetamine* OR

anxiolytic* OR barbiturate* OR benzo* OR cocaine OR ethanol OR fentanyl OR (glue adj1 sniff*) OR heroin or huffing* OR hypnotic* OR inhalant* OR meth OR methamphetamine* OR morphine OR narcotic* OR opioid* OR opiate* OR opium OR PCP OR phencyclidine OR sedative* OR stimulant*) adj5 (addict* OR dependen*)).ti,ab. OR ((drug* OR substance*) ADJ3 (addict* OR dependen*)).ti,ab,kf. OR ((alcohol or amphetamine* OR anxiolytic* OR barbiturate* OR benzo* OR cocaine OR ethanol OR fentanyl OR (glue adj1 sniff*) OR heroin or huffing* OR hypnotic* OR inhalant* OR meth OR methamphetamine* OR morphine OR narcotic* OR opioid* OR opiate* OR opium OR PCP OR phencyclidine OR sedative* OR stimulant*) AND (dependen*)).kf. OR use-disorder*.ti,ab,hw,kf,dq.)**)** **AND** **(**(cannabinoid/dt OR cannabis/dt OR ketamine/dt OR medical cannabis/dt OR nitrous oxide/dt OR exp psychedelic agent/dt OR psychedelic therapy/ OR (((medical OR medicin*) ADJ2 (cannabis OR mari#uana)) OR ((cannabinoid* OR cannabis OR DMT OR dimethyltryptamine OR hallucinogen* OR ketamine OR LSD OR lysergic-acid-diethylamide OR mari#uana OR MDMA OR methylenedioxymethamphetamine OR nitrous-oxide OR psilocybin* OR psych#delic*) ADJ3 (pharmacotherap* OR therap* OR treat*))).ti,ab,kf,dq.) OR (brain depth stimulation/ OR exp focused ultrasound therapy/ OR transcranial direct current stimulation/ OR exp transcranial magnetic stimulation/ OR (brain-depth-stimulation OR DBS OR DCS OR deep-brain-stimulation OR direct-current-stimulation OR focused-ultrasound OR FUS OR TMS OR transcranial-magnetic-stimulation).ti,ab,kf.) OR ((interventional) ADJ3 (psychiatr*)).ti,ab,kf,dq.**))** **OR** **((**(cannabis addiction/ OR cannab*-use-disorder*.ti,ab,kf. OR ((cannabinoid* OR cannabis OR mari#uana) ADJ5 (addict* OR dependen*)).ti,ab. OR ((cannabinoid* OR cannabis OR mari#uana) AND (addict* OR dependen*)).kf.)**)** **AND** **(**(brain depth stimulation/ OR exp focused ultrasound therapy/ OR transcranial direct current stimulation/ OR exp transcranial magnetic stimulation/ OR (brain-depth-stimulation OR DBS OR DCS OR deep-brain-stimulation OR direct-current-stimulation OR focused-ultrasound OR FUS OR TMS OR transcranial-magnetic-stimulation).ti,ab,kf.) OR ((interventional) ADJ3 (psychiatr*)).ti,ab,kf,dq.**))** **AND** **(**((Systematic OR metaanalysis OR meta-analysis) ADJ (review)).ti,ab OR systematic review.pt OR meta analysis.pt OR (Cochrane OR Embase OR MEDLINE OR PubMed OR Scopus OR PRISMA).ab.**)** **AND** **(**English.la**)** **NOT** (conference abstract OR conference review).pt.

Embase 1974 to 2025 April 01 (Wolters Kluwer Ovid interface)

Date searched: April 02, 2025

Records retrieved: 49

Language: limited to English

Study design: limited to systematic review with or without meta-analysis

Publication type: excluded conference abstract and conference review

*This search reflects additional interventions: electroconvulsive therapy and vagal nerve stimulation.

((alcoholism/ OR amphetamine dependence/ OR benzodiazepine dependence/ OR cocaine dependence/ OR drug dependence/ OR methamphetamine dependence/ OR exp narcotic dependence/ OR phencyclidine dependence/ OR alcoholism.ti,ab,kf. OR (addict OR addicts OR alcoholic*).ti,kf OR ((alcohol or amphetamine* OR anxiolytic* OR barbiturate* OR benzo* OR cocaine OR ethanol OR fentanyl OR (glue adj1 sniff*) OR heroin or huffing* OR hypnotic* OR inhalant* OR meth OR methamphetamine* OR morphine OR narcotic* OR opioid* OR opiate* OR opium OR PCP OR phencyclidine OR sedative* OR stimulant*) adj5 (addict* OR dependen*)).ti,ab. OR ((drug* OR substance*) ADJ3 (addict* OR dependen*)).ti,ab,kf. OR ((alcohol or amphetamine* OR anxiolytic* OR barbiturate* OR benzo* OR cocaine OR ethanol OR fentanyl OR (glue adj1 sniff*) OR heroin or huffing* OR hypnotic* OR inhalant* OR meth OR methamphetamine* OR morphine OR narcotic* OR opioid* OR opiate* OR opium OR PCP OR phencyclidine OR sedative* OR stimulant*) AND (dependen*)).kf. OR use-disorder*.ti,ab,hw,kf,dq.) **OR** (cannabis addiction/ OR cannab*-use-disorder*.ti,ab,kf. OR ((cannabinoid* OR cannabis OR mari#uana) ADJ5 (addict* OR dependen*)).ti,ab. OR ((cannabinoid* OR cannabis OR mari#uana) AND (addict* OR dependen*)).kf.)) **AND** (Electroconvulsive therapy/ OR vagus nerve stimulation/ OR (ECT OR electroconvuls* OR electr*-convuls* OR electroshock* OR electr*-shock* OR VNS OR ((vagal OR vagus) ADJ5 (stimulat*))).ti,ab,kf.) **AND** (Exp meta analysis/ OR systematic review/ OR (metaanalysis OR meta-analysis OR systematic review).ti,ab,kw OR (Cochrane OR Embase OR MEDLINE OR PubMed OR Scopus OR PRISMA).ab.) **AND** english.la **NOT** (conference abstract OR conference review).pt.

Ovid MEDLINE(R) 1946 to Present and Epub Ahead of Print, In-Process & Other Non-Indexed Citations and Ovid MEDLINE(R) Daily

Date searched: December 20, 2024

Records retrieved: 247

Language: limited to English

Study design: limited to systematic review with OR without meta-analysis

(((alcoholism/ OR amphetamine-related disorders/ OR cocaine-related disorders/ OR narcotic-related disorders/ OR opioid-related disorders/ OR substance-related disorders/ OR alcoholism.ti,ab,kf. OR (addict OR addicts OR alcoholic*).ti,kf OR ((alcohol or amphetamine* OR anxiolytic* OR barbiturate* OR benzo* OR cocaine OR ethanol OR fentanyl OR (glue adj1 sniff*) OR heroin or huffing* OR hypnotic*

OR inhalant* OR meth OR methamphetamine* OR morphine OR narcotic* OR opioid* OR opiate* OR opium OR PCP OR phencyclidine OR sedative* OR stimulant*) adj5 (addict* OR dependen*)).ti,ab. OR ((drug* OR substance*) ADJ3 (addict* OR dependen*)).ti,ab,kf. OR ((alcohol or amphetamine* OR anxiolytic* OR barbiturate* OR benzo* OR cocaine OR ethanol OR fentanyl OR (glue adj1 sniff*) OR heroin or huffing* OR hypnotic* OR inhalant* OR meth OR methamphetamine* OR morphine OR narcotic* OR opioid* OR opiate* OR opium OR PCP OR phencyclidine OR sedative* OR stimulant*) AND (dependen*)).kf. OR use-disorder*.ti,ab,hw,kf.)**) AND (**(cannabinoids/tu OR cannabis/tu OR exp hallucinogens/tu OR ketamine/tu OR medical marijuana/tu OR nitrous oxide/tu OR (((medical OR medicin*) ADJ2 (cannabis OR mari#uana)) OR ((cannabinoid* OR cannabis OR DMT OR dimethyltryptamine OR hallucinogen* OR ketamine OR LSD OR lysergic-acid-diethylamide OR mari#uana OR MDMA OR methylenedioxymethamphetamine OR nitrous-oxide OR psilocybin* OR psych#delic*) ADJ3 (pharmacotherap* OR therap* OR treat*))).ti,ab,kf.) OR (deep brain stimulation/ OR transcranial direct current stimulation/ OR exp transcranial magnetic stimulation/ OR exp ultrasonic therapy/ OR (brain-depth-stimulation OR DBS OR DCS OR deep-brain-stimulation OR direct-current-stimulation OR focused-ultrasound OR FUS OR TMS OR transcranial-magnetic-stimulation).ti,ab,kf.) OR ((interventional) ADJ3 (psychiatr*)).ti,ab,kf.**)) OR ((**cannab*-use-disorder*.ti,ab,kf. OR ((cannabinoid* OR cannabis OR mari#uana) ADJ5 (addict* OR dependen*)).ti,ab. OR ((cannabinoid* OR cannabis OR mari#uana) AND (addict* OR dependen*)).kf.**) AND (**(deep brain stimulation/ OR transcranial direct current stimulation/ OR exp transcranial magnetic stimulation/ OR exp ultrasonic therapy/ OR (brain-depth-stimulation OR DBS OR DCS OR deep-brain-stimulation OR direct-current-stimulation OR focused-ultrasound OR FUS OR TMS OR transcranial-magnetic-stimulation).ti,ab,kf.) OR ((interventional) ADJ3 (psychiatr*)).ti,ab,kf.**)) AND (**((Systematic OR metaanalysis OR meta-analysis) ADJ (review)).ti,ab OR systematic review.pt OR meta analysis.pt OR (Cochrane OR Embase OR MEDLINE OR PubMed OR Scopus OR PRISMA).ab.**) AND** (English.la)

Ovid MEDLINE(R) 1946 to Present and Epub Ahead of Print, In-Process & Other Non-Indexed Citations and Ovid MEDLINE(R) Daily

Date searched: April 02, 2025

Records retrieved: 15

Language: limited to English

Study design: limited to systematic review with OR without meta-analysis

*This search reflects additional interventions: electroconvulsive therapy and vagal nerve stimulation.

((alcoholism/ OR amphetamine-related disorders/ OR cocaine-related disorders/ OR narcotic-related disorders/ OR opioid-related disorders/ OR substance-related disorders/ OR alcoholism.ti,ab,kf. OR (addict OR addicts OR alcoholic*).ti,kf OR ((alcohol or amphetamine* OR anxiolytic* OR barbiturate* OR benzo* OR cocaine OR ethanol OR fentanyl OR (glue adj1 sniff*) OR heroin or huffing* OR hypnotic* OR inhalant* OR meth OR methamphetamine* OR morphine OR narcotic* OR opioid* OR opiate* OR opium OR PCP OR phencyclidine OR sedative* OR stimulant*) adj5 (addict* OR dependen*)).ti,ab. OR ((drug* OR substance*) ADJ3 (addict* OR dependen*)).ti,ab,kf. OR ((alcohol or amphetamine* OR anxiolytic* OR barbiturate* OR benzo* OR cocaine OR ethanol OR fentanyl OR (glue adj1 sniff*) OR heroin or huffing* OR hypnotic* OR inhalant* OR meth OR methamphetamine* OR morphine OR narcotic* OR opioid* OR opiate* OR opium OR PCP OR phencyclidine OR sedative* OR stimulant*) AND (dependen*)).kf. OR use-disorder*.ti,ab,hw,kf.) **OR** cannab*-use-disorder*.ti,ab,kf. OR ((cannabinoid* OR cannabis OR mari#uana) ADJ5 (addict* OR dependen*)).ti,ab. OR ((cannabinoid* OR cannabis OR mari#uana) AND (addict* OR dependen*)).kf.) **AND** (Electroconvulsive therapy/ OR vagus nerve stimulation/ OR (ECT OR electroconvuls* OR electr*-convuls* OR electroshock* OR electr*-shock* OR VNS OR ((vagal OR vagus) ADJ5 (stimulat*))).ti,ab,kf.) **AND** ((meta-analysis OR systematic review).pt,ti,ab OR (Cochrane OR Embase OR MEDLINE OR PubMed OR Scopus OR PRISMA).ab.) **AND** english.la

APA PsycINFO 1806 to December 2024 Week 3 (Wolters Kluwer Ovid interface)

Date searched: December 20, 2024

Records retrieved: 104

Language: limited to English

Study design: limited to systematic review with or without meta-analysis

(((alcoholism/ OR “alcohol use disorder”/ OR drug addiction/ OR drug dependency/ OR exp “opioid use disorder”/ OR “substance use disorder”/ OR alcoholism.ti,ab,id. OR (addict OR addicts OR alcoholic*).ti,id OR ((alcohol or amphetamine* OR anxiolytic* OR barbiturate* OR benzo* OR cocaine OR ethanol OR fentanyl OR (glue adj1 sniff*) OR heroin or huffing* OR hypnotic* OR inhalant* OR meth OR methamphetamine* OR morphine OR narcotic* OR opioid* OR opiate* OR opium OR PCP OR phencyclidine OR sedative* OR stimulant*) adj5 (addict* OR dependen*)).ti,ab. OR ((drug* OR substance*) ADJ3 (addict* OR dependen*)).ti,ab,id. OR ((alcohol or amphetamine* OR anxiolytic* OR barbiturate* OR benzo* OR cocaine OR ethanol OR fentanyl OR (glue adj1 sniff*) OR heroin or huffing* OR hypnotic* OR inhalant* OR meth OR methamphetamine* OR morphine OR narcotic* OR opioid* OR opiate* OR opium OR PCP OR phencyclidine OR sedative* OR stimulant*) AND

(dependen*)).id. OR use-disorder*.ti,ab,hw,id.)) **AND** ((medical marijuana/ OR psychedelic assisted therapy/ OR (((medical OR medicin*) ADJ2 (cannabis OR mari#uana)) OR ((cannabinoid* OR cannabis OR DMT OR dimethyltryptamine OR hallucinogen* OR ketamine OR LSD OR lysergic-acid-diethylamide OR mari#uana OR MDMA OR methylenedioxymethamphetamine OR nitrous-oxide OR psilocybin* OR psych#delic*) ADJ3 (pharmacotherap* OR therap* OR treat*))).ti,ab,id.) OR (deep brain stimulation/ OR transcranial direct current stimulation/ OR exp transcranial magnetic stimulation/ OR (brain-depth-stimulation OR DBS OR DCS OR deep-brain-stimulation OR direct-current-stimulation OR focused-ultrasound OR FUS OR TMS OR transcranial-magnetic-stimulation).ti,ab,id.) OR ((interventional) ADJ3 (psychiatr*)).ti,ab,id.)) **OR** (((“cannabis use disorder”/ OR cannab*-use-disorder*.ti,ab,id. OR ((cannabinoid* OR cannabis OR mari#uana) ADJ5 (addict* OR dependen*)).ti,ab. OR ((cannabinoid* OR cannabis OR mari#uana) AND (addict* OR dependen*)).id.)) **AND** ((deep brain stimulation/ OR transcranial direct current stimulation/ OR exp transcranial magnetic stimulation/ OR (brain-depth-stimulation OR DBS OR DCS OR deep-brain-stimulation OR direct-current-stimulation OR focused-ultrasound OR FUS OR TMS OR transcranial-magnetic-stimulation).ti,ab,id.) OR ((interventional) ADJ3 (psychiatr*)).ti,ab,id.)) **AND** (((Systematic OR metaanalysis OR meta-analysis) ADJ (review)).ti,ab OR systematic review.pt OR meta analysis.pt OR (Cochrane OR Embase OR MEDLINE OR PubMed OR Scopus OR PRISMA).ab.) **AND** (English.la)

APA PsycINFO 1806 to March 2025 Week 4 (Wolters Kluwer Ovid interface)

Date searched: April 02, 2025

Records retrieved: 7

Language: limited to English

Study design: limited to systematic review with or without meta-analysis

*This search reflects additional interventions: electroconvulsive therapy and vagal nerve stimulation.

((alcoholism/ OR “alcohol use disorder”/ OR drug addiction/ OR drug dependency/ OR exp “opioid use disorder”/ OR “substance use disorder”/ OR alcoholism.ti,ab,id. OR (addict OR addicts OR alcoholic*).ti,id OR ((alcohol or amphetamine* OR anxiolytic* OR barbiturate* OR benzo* OR cocaine OR ethanol OR fentanyl OR (glue adj1 sniff*) OR heroin or huffing* OR hypnotic* OR inhalant* OR meth OR methamphetamine* OR morphine OR narcotic* OR opioid* OR opiate* OR opium OR PCP OR phencyclidine OR sedative* OR stimulant*) adj5 (addict* OR dependen*)).ti,ab. OR ((drug* OR substance*) ADJ3 (addict* OR dependen*)).ti,ab,id. OR ((alcohol or amphetamine* OR anxiolytic* OR

barbiturate* OR benzo* OR cocaine OR ethanol OR fentanyl OR (glue adj1 sniff*) OR heroin or huffing* OR hypnotic* OR inhalant* OR meth OR methamphetamine* OR morphine OR narcotic* OR opioid* OR opiate* OR opium OR PCP OR phencyclidine OR sedative* OR stimulant*) AND (dependen*)).id. OR use-disorder*.ti,ab,hw,id.) **OR** (“cannabis use disorder”/ OR cannab*-use-disorder*.ti,ab,id. OR ((cannabinoid* OR cannabis OR mari#uana) ADJ5 (addict* OR dependen*)).ti,ab. OR ((cannabinoid* OR cannabis OR mari#uana) AND (addict* OR dependen*)).id.)) **AND** (Electroconvulsive shock therapy/ OR ((nerve stimulation/) AND (vagus nerve/ OR (vagal OR vagus).ti)) OR (ECT OR electroconvuls* OR electr*-convuls* OR electroshock* OR electr*-shock* OR VNS OR ((vagal OR vagus) ADJ5 (stimulat*))).ti,ab,id.) **AND** (Meta analysis/ OR Systematic review/ OR (metaanalysis OR meta-analysis OR systematic review).pt,ti,ab,id OR (Cochrane OR Embase OR MEDLINE OR PubMed OR Scopus OR PRISMA).ab.) **AND** english.la

EBM Reviews - Cochrane Database of Systematic Reviews 2005 to December 18, 2024 (Wolters Kluwer Ovid interface)

Date searched: December 20, 2024

Records retrieved: 5

((alcoholism.ti,ab,kw. OR (addict OR addicts OR alcoholic*).ti,kw OR ((alcohol or amphetamine* OR anxiolytic* OR barbiturate* OR benzo* OR cocaine OR ethanol OR fentanyl OR (glue adj1 sniff*) OR heroin or huffing* OR hypnotic* OR inhalant* OR meth OR methamphetamine* OR morphine OR narcotic* OR opioid* OR opiate* OR opium OR PCP OR phencyclidine OR sedative* OR stimulant*) adj5 (addict* OR dependen*)).ti,ab. OR ((drug* OR substance*) ADJ3 (addict* OR dependen*)).ti,ab,kw. OR ((alcohol or amphetamine* OR anxiolytic* OR barbiturate* OR benzo* OR cocaine OR ethanol OR fentanyl OR (glue adj1 sniff*) OR heroin or huffing* OR hypnotic* OR inhalant* OR meth OR methamphetamine* OR morphine OR narcotic* OR opioid* OR opiate* OR opium OR PCP OR phencyclidine OR sedative* OR stimulant*) AND (dependen*)).kw. OR use-disorder*.ti,ab,kw.**)** **AND** **(**(((medical OR medicin*) ADJ2 (cannabis OR mari#uana)) OR ((cannabinoid* OR cannabis OR DMT OR dimethyltryptamine OR hallucinogen* OR ketamine OR LSD OR lysergic-acid-diethylamide OR mari#uana OR MDMA OR methylenedioxymethamphetamine OR nitrous-oxide OR psilocybin* OR psych#delic*) ADJ3 (pharmacotherap* OR therap* OR treat*))).ti,ab,kw. OR (brain-depth-stimulation OR DBS OR DCS OR deep-brain-stimulation OR direct-current-stimulation OR focused-ultrasound OR FUS OR TMS OR transcranial-magnetic-stimulation).ti,ab,kw. OR ((interventional) ADJ3 (psychiatr*)).ti,ab,kw**))** **OR** **((**cannab*-use-disorder*.ti,ab,kw. OR ((cannabinoid* OR cannabis OR mari#uana) ADJ5 (addict* OR dependen*)).ti,ab. OR ((cannabinoid* OR cannabis OR mari#uana) AND (addict* OR dependen*)).kw.**)** **AND**

((brain-depth-stimulation OR DBS OR DCS OR deep-brain-stimulation OR direct-current-stimulation OR focused-ultrasound OR FUS OR TMS OR transcranial-magnetic-stimulation).ti,ab,kw. OR ((interventional) ADJ3 (psychiatr*)).ti,ab,kw**)**

EBM Reviews - Cochrane Database of Systematic Reviews 2005 to March 26, 2025 (Wolters Kluwer Ovid interface)

Date searched: April 02, 2025

Records retrieved: 0

*This search reflects additional interventions: electroconvulsive therapy and vagal nerve stimulation.

(alcoholism.ti,ab,kw. OR (addict OR addicts OR alcoholic*).ti,kw OR ((alcohol or amphetamine* OR anxiolytic* OR barbiturate* OR benzo* OR cocaine OR ethanol OR fentanyl OR (glue adj1 sniff*) OR heroin or huffing* OR hypnotic* OR inhalant* OR meth OR methamphetamine* OR morphine OR narcotic* OR opioid* OR opiate* OR opium OR PCP OR phencyclidine OR sedative* OR stimulant*) adj5 (addict* OR dependen*)).ti,ab. OR ((drug* OR substance*) ADJ3 (addict* OR dependen*)).ti,ab,kw. OR ((alcohol or amphetamine* OR anxiolytic* OR barbiturate* OR benzo* OR cocaine OR ethanol OR fentanyl OR (glue adj1 sniff*) OR heroin or huffing* OR hypnotic* OR inhalant* OR meth OR methamphetamine* OR morphine OR narcotic* OR opioid* OR opiate* OR opium OR PCP OR phencyclidine OR sedative* OR stimulant*) AND (dependen*)).kw. OR use-disorder*.ti,ab,kw. **OR** cannab*-use-disorder*.ti,ab,kw. OR ((cannabinoid* OR cannabis OR mari#uana) ADJ5 (addict* OR dependen*)).ti,ab. OR ((cannabinoid* OR cannabis OR mari#uana) AND (addict* OR dependen*)).kw.) **AND** (ECT OR electroconvuls* OR electr*-convuls* OR electroshock* OR electr*-shock* OR VNS OR ((vagal OR vagus) ADJ5 (stimulat*))).ti,ab,kw

SCOPUS

Date searched: December 20, 2024

Records retrieved: 296

Language: limited to English

Subject Areas: limited to Medicine; Neuroscience; Pharmacology, Toxicology and Pharmaceutics; and Psychology

((TITLE-ABS-KEY (alcoholism) OR TITLE (addict OR addicts OR alcoholic) OR KEY (addict OR addicts OR alcoholic) OR TITLE-ABS ((alcohol or amphetamine OR anxiolytic OR

barbiturate OR benzo OR cocaine OR ethanol OR fentanyl OR (glue W/1 sniff*) OR heroin or huffing OR hypnotic OR inhalant OR meth OR methamphetamine OR morphine OR narcotic OR opioid OR opiate OR opium OR PCP OR phencyclidine OR sedative OR stimulant) W/4 (addict* OR dependen*)) OR TITLE-ABS-KEY ((drug* OR substance*) W/2 (addict* OR dependen*)) OR KEY ((alcohol or amphetamine OR anxiolytic OR barbiturate OR benzo OR cocaine OR ethanol OR fentanyl OR (glue W/1 sniff*) OR heroin or huffing OR hypnotic OR inhalant OR meth OR methamphetamine OR morphine OR narcotic OR opioid OR opiate OR opium OR PCP OR phencyclidine OR sedative OR stimulant) AND (dependen*)) OR TITLE-ABS-KEY (use-disorder)**) AND (**TITLE-ABS-KEY (((medical OR medicin*) W/1 (cannabis OR marijuana)) OR ((cannabinoid OR cannabis OR DMT OR dimethyltryptamine OR hallucinogen* OR ketamine OR LSD OR lysergic-acid-diethylamide OR marijuana OR MDMA OR methylenedioxymethamphetamine OR nitrous-oxide OR psilocybin OR psychedelic) W/3 (pharmacotherap* OR therap* OR treat*))) OR TITLE-ABS-KEY (brain-depth-stimulation OR deep-brain-stimulation OR direct-current-stimulation OR focused-ultrasound OR transcranial-magnetic-stimulation) OR Title-ABS-KEY ((interventional) W/2 (psychiatr*))**)) OR ((**TITLE-ABS-KEY (cannabis-use-disorder OR ((cannabinoid OR cannabis OR marijuana) W/4 (addict* OR dependen*)) OR ((cannabinoid OR cannabis OR marijuana) AND (addict* OR dependen*)))**) AND (**TITLE-ABS-KEY (brain-depth-stimulation OR deep-brain-stimulation OR direct-current-stimulation OR focused-ultrasound OR transcranial-magnetic-stimulation) OR Title-ABS-KEY ((interventional) W/2 (psychiatr*))**)) AND** (TITLE-ABS ((Systematic OR metaanalysis OR meta-analysis) PRE/0 (review)) OR ABS (Cochrane OR Embase OR MEDLINE OR PubMed OR Scopus OR PRISMA)) AND (LIMIT-TO (LANGUAGE , "English")) AND (LIMIT-TO (LANGUAGE, "English")) AND (LIMIT-TO (SUBJAREA, "MEDI") OR LIMIT-TO (SUBJAREA, "NEUR") OR LIMIT-TO (SUBJAREA, "PHAR") OR LIMIT-TO (SUBJAREA, "PSYC"))

SCOPUS

Date searched: April 02, 2025

Records retrieved: 53

Language: limited to English

Subject Areas: limited to Medicine; Neuroscience; Pharmacology, Toxicology and Pharmaceutics; and Psychology

*This search reflects additional interventions: electroconvulsive therapy and vagal nerve stimulation.

(TITLE-ABS-KEY (alcoholism) OR TITLE (addict OR addicts OR alcoholic) OR KEY (addict OR addicts OR alcoholic) OR TITLE-ABS ((alcohol or amphetamine OR anxiolytic OR barbiturate OR benzo OR cocaine OR ethanol OR fentanyl OR (glue

W/1 sniff*) OR heroin or huffing OR hypnotic OR inhalant OR meth OR methamphetamine OR morphine OR narcotic OR opioid OR opiate OR opium OR PCP OR phencyclidine OR sedative OR stimulant) W/4 (addict* OR dependen*)) OR TITLE-ABS-KEY ((drug* OR substance*) W/2 (addict* OR dependen*)) OR KEY ((alcohol or amphetamine OR anxiolytic OR barbiturate OR benzo OR cocaine OR ethanol OR fentanyl OR (glue W/1 sniff*) OR heroin or huffing OR hypnotic OR inhalant OR meth OR methamphetamine OR morphine OR narcotic OR opioid OR opiate OR opium OR PCP OR phencyclidine OR sedative OR stimulant) AND (dependen*)) OR TITLE-ABS-KEY (use-disorder) **OR** TITLE-ABS-KEY (cannabis-use-disorder OR ((cannabinoid OR cannabis OR marijuana) W/4 (addict* OR dependen*)) OR ((cannabinoid OR cannabis OR marijuana) AND (addict* OR dependen*)))) **AND** TITLE-ABS-KEY (ECT OR electroconvuls* OR electr*-convuls* OR electroshock* OR electr*-shock* OR VNS OR ((vagal OR vagus) W/4 (stimulat*))) **AND** TITLE-ABS (metaanalysis OR meta-analysis OR systematic-review) OR ABS (Cochrane OR Embase OR MEDLINE OR PubMed OR Scopus OR PRISMA) **AND** (LIMIT-TO (SUBJAREA , "MEDI") OR LIMIT-TO (SUBJAREA , "NEUR") OR LIMIT-TO (SUBJAREA , "PHAR") OR LIMIT-TO (SUBJAREA , "PSYC")) AND (LIMIT-TO (LANGUAGE , "English"))

Science Citation Index Expanded (SCI-Expanded)—1975-present (Web of Science)

Date searched: December 20, 2024

Records retrieved: 193

Language: limited to English

Categories: limited to Behavioral Sciences; Clinical Neurology; Medicine General Internal; Neurosciences; Pharmacology Pharmacy; Psychiatry; Psychology; Psychology Clinical; Substance Abuse.

Document Types: excluded meeting abstracts

(((TS=(alcoholism) OR TI=(addict OR addicts OR alcoholic) OR AK=(addict OR addicts OR alcoholic) OR TI=((alcohol or amphetamine OR anxiolytic OR barbiturate OR benzo OR cocaine OR ethanol OR fentanyl OR (glue NEAR/1 sniff*) OR heroin or huffing OR hypnotic OR inhalant OR meth OR methamphetamine OR morphine OR narcotic OR opioid OR opiate OR opium OR PCP OR phencyclidine OR sedative OR stimulant) NEAR/4 (addict* OR dependen*)) OR AB=((alcohol or amphetamine OR anxiolytic OR barbiturate OR benzo OR cocaine OR ethanol OR fentanyl OR (glue NEAR/1 sniff*) OR heroin or huffing OR hypnotic OR inhalant OR meth OR methamphetamine OR morphine OR narcotic OR opioid OR opiate OR opium OR PCP OR phencyclidine OR sedative OR stimulant) NEAR/4 (addict* OR dependen*)) OR TS=((drug* OR substance*) NEAR/2 (addict* OR dependen*)) OR AK=((alcohol or

amphetamine OR anxiolytic OR barbiturate OR benzo OR cocaine OR ethanol OR fentanyl OR (glue W/0 sniff*) OR heroin or huffing OR hypnotic OR inhalant OR meth OR methamphetamine OR morphine OR narcotic OR opioid OR opiate OR opium OR PCP OR phencyclidine OR sedative OR stimulant) AND (dependen*)) OR TS=(use-disorder)**)** **AND** **(**TS=(((medical OR medicin*) NEAR/1 (cannabis OR marijuana)) OR ((cannabinoid OR cannabis OR DMT OR dimethyltryptamine OR hallucinogen* OR ketamine OR LSD OR lysergic-acid-diethylamide OR marijuana OR MDMA OR methylenedioxymethamphetamine OR nitrous-oxide OR psilocybin OR psychedelic) NEAR/3 (pharmacotherap* OR therap* OR treat*))) OR TS=(brain-depth-stimulation OR deep-brain-stimulation OR direct-current-stimulation OR focused-ultrasound OR transcranial-magnetic-stimulation) OR TS=((interventional) NEAR/2 (psychiatr*))**))** **OR** **((**TS=(cannabis-use-disorder OR ((cannabinoid OR cannabis OR marijuana) NEAR/4 (addict* OR dependen*)) OR ((cannabinoid OR cannabis OR marijuana) AND (addict* OR dependen*)))**)** AND **(**TS=(brain-depth-stimulation OR deep-brain-stimulation OR direct-current-stimulation OR focused-ultrasound OR transcranial-magnetic-stimulation) OR TS=((interventional) NEAR/2 (psychiatr*))**)))** **AND** **(**TI=((Systematic OR metaanalysis OR meta-analysis) NEAR/0 (review)) OR AB=(Cochrane OR Embase OR MEDLINE OR PubMed OR Scopus OR PRISMA OR ((Systematic OR metaanalysis OR meta-analysis) NEAR/0 (review)))**)**

Science Citation Index Expanded (SCI-Expanded)—1975-present (Web of Science)

Date searched: April 02, 2025

Records retrieved: 15

Language: limited to English

Categories: limited to Behavioral Sciences; Clinical Neurology; Medicine General Internal; Neurosciences; Pharmacology Pharmacy; Psychiatry; Psychology; Psychology Clinical; Substance Abuse.

Document Types: excluded meeting abstracts

*This search reflects additional interventions: electroconvulsive therapy and vagal nerve stimulation.

(TS=(alcoholism) OR TI=(addict OR addicts OR alcoholic) OR AK=(addict OR addicts OR alcoholic) OR TI=((alcohol or amphetamine OR anxiolytic OR barbiturate OR benzo OR cocaine OR ethanol OR fentanyl OR (glue NEAR/1 sniff*) OR heroin or huffing OR hypnotic OR inhalant OR meth OR methamphetamine OR morphine OR narcotic OR opioid OR opiate OR opium OR PCP OR phencyclidine OR sedative OR stimulant) NEAR/4 (addict* OR dependen*)) OR AB=((alcohol or amphetamine OR

anxiolytic OR barbiturate OR benzo OR cocaine OR ethanol OR fentanyl OR (glue NEAR/1 sniff*) OR heroin or huffing OR hypnotic OR inhalant OR meth OR methamphetamine OR morphine OR narcotic OR opioid OR opiate OR opium OR PCP OR phencyclidine OR sedative OR stimulant) NEAR/4 (addict* OR dependen*)) OR TS=((drug* OR substance*) NEAR/2 (addict* OR dependen*)) OR AK=((alcohol or amphetamine OR anxiolytic OR barbiturate OR benzo OR cocaine OR ethanol OR fentanyl OR (glue W/0 sniff*) OR heroin or huffing OR hypnotic OR inhalant OR meth OR methamphetamine OR morphine OR narcotic OR opioid OR opiate OR opium OR PCP OR phencyclidine OR sedative OR stimulant) AND (dependen*)) OR TS=(use-disorder) OR TS=(cannabis-use-disorder OR ((cannabinoid OR cannabis OR marijuana) NEAR/4 (addict* OR dependen*)) OR ((cannabinoid OR cannabis OR marijuana) AND (addict* OR dependen*)))) AND (TS=(ECT OR electroconvuls* OR electr*-convuls* OR electroshock* OR electr*-shock* OR VNS OR ((vagal OR vagus) NEAR/4 (stimulat*)))) **AND (**TI=(Systematic OR metaanalysis OR meta-analysis) OR AB=(Systematic OR metaanalysis OR meta-analysis OR Cochrane OR Embase OR MEDLINE OR PubMed OR Scopus OR PRISMA**))**

Emerging Sources Citation Index (ESCI)—2018-present (Web of Science)

Date searched: December 20, 2024

Records retrieved: 17

Language: limited to English

Categories: limited to Clinical Neurology; Medicine General Internal; Neurosciences; Pharmacology Pharmacy; Psychiatry; Psychology Clinical; and Substance Abuse.

(((TS=(alcoholism) OR TI=(addict OR addicts OR alcoholic) OR AK=(addict OR addicts OR alcoholic) OR TI=((alcohol or amphetamine OR anxiolytic OR barbiturate OR benzo OR cocaine OR ethanol OR fentanyl OR (glue NEAR/1 sniff*) OR heroin or huffing OR hypnotic OR inhalant OR meth OR methamphetamine OR morphine OR narcotic OR opioid OR opiate OR opium OR PCP OR phencyclidine OR sedative OR stimulant) NEAR/4 (addict* OR dependen*)) OR AB=((alcohol or amphetamine OR anxiolytic OR barbiturate OR benzo OR cocaine OR ethanol OR fentanyl OR (glue NEAR/1 sniff*) OR heroin or huffing OR hypnotic OR inhalant OR meth OR methamphetamine OR morphine OR narcotic OR opioid OR opiate OR opium OR PCP OR phencyclidine OR sedative OR stimulant) NEAR/4 (addict* OR dependen*)) OR TS=((drug* OR substance*) NEAR/2 (addict* OR dependen*)) OR AK=((alcohol or amphetamine OR anxiolytic OR barbiturate OR benzo OR cocaine OR ethanol OR fentanyl OR (glue W/0 sniff*) OR heroin or huffing OR hypnotic OR inhalant OR meth OR methamphetamine OR morphine OR narcotic OR opioid OR opiate OR opium OR PCP OR phencyclidine OR sedative OR stimulant) AND (dependen*)) OR TS=(use-disorder)**) AND (**TS=(((medical OR medicin*) NEAR/1 (cannabis OR

marijuana)) OR ((cannabinoid OR cannabis OR DMT OR dimethyltryptamine OR hallucinogen* OR ketamine OR LSD OR lysergic-acid-diethylamide OR marijuana OR MDMA OR methylenedioxymethamphetamine OR nitrous-oxide OR psilocybin OR psychedelic) NEAR/3 (pharmacotherap* OR therap* OR treat*))) OR TS=(brain-depth-stimulation OR deep-brain-stimulation OR direct-current-stimulation OR focused-ultrasound OR transcranial-magnetic-stimulation) OR TS=((interventional) NEAR/2 (psychiatr*))**)) OR ((**TS=(cannabis-use-disorder OR ((cannabinoid OR cannabis OR marijuana) NEAR/4 (addict* OR dependen*)) OR ((cannabinoid OR cannabis OR marijuana) AND (addict* OR dependen*))))**)** AND **(**TS=(brain-depth-stimulation OR deep-brain-stimulation OR direct-current-stimulation OR focused-ultrasound OR transcranial-magnetic-stimulation) OR TS=((interventional) NEAR/2 (psychiatr*))**))) AND (**TI=((Systematic OR metaanalysis OR meta-analysis) NEAR/0 (review)) OR AB=(Cochrane OR Embase OR MEDLINE OR PubMed OR Scopus OR PRISMA OR ((Systematic OR metaanalysis OR meta-analysis) NEAR/0 (review)))**)**

Emerging Sources Citation Index (ESCI)—2018-present (Web of Science)

Date searched: April 02, 2025

Records retrieved: 1

Language: limited to English

Categories: limited to Clinical Neurology; Medicine General Internal; Neurosciences; Pharmacology Pharmacy; Psychiatry; Psychology Clinical; and Substance Abuse.

*This search reflects additional interventions: electroconvulsive therapy and vagal nerve stimulation.

(TS=(alcoholism) OR TI=(addict OR addicts OR alcoholic) OR AK=(addict OR addicts OR alcoholic) OR TI=((alcohol or amphetamine OR anxiolytic OR barbiturate OR benzo OR cocaine OR ethanol OR fentanyl OR (glue NEAR/1 sniff*) OR heroin or huffing OR hypnotic OR inhalant OR meth OR methamphetamine OR morphine OR narcotic OR opioid OR opiate OR opium OR PCP OR phencyclidine OR sedative OR stimulant) NEAR/4 (addict* OR dependen*)) OR AB=((alcohol or amphetamine OR anxiolytic OR barbiturate OR benzo OR cocaine OR ethanol OR fentanyl OR (glue NEAR/1 sniff*) OR heroin or huffing OR hypnotic OR inhalant OR meth OR methamphetamine OR morphine OR narcotic OR opioid OR opiate OR opium OR PCP OR phencyclidine OR sedative OR stimulant) NEAR/4 (addict* OR dependen*)) OR TS=((drug* OR substance*) NEAR/2 (addict* OR dependen*)) OR AK=((alcohol or amphetamine OR anxiolytic OR barbiturate OR benzo OR cocaine OR ethanol OR fentanyl OR (glue W/0 sniff*) OR heroin or huffing OR hypnotic OR inhalant OR meth OR methamphetamine OR morphine OR narcotic OR opioid OR opiate OR opium OR PCP OR phencyclidine OR sedative OR stimulant) AND (dependen*)) OR

TS=(use-disorder) OR TS=(cannabis-use-disorder OR ((cannabinoid OR cannabis OR marijuana) NEAR/4 (addict* OR dependen*)) OR ((cannabinoid OR cannabis OR marijuana) AND (addict* OR dependen*)))) AND (TS=(ECT OR electroconvuls* OR electr*-convuls* OR electroshock* OR electr*-shock* OR VNS OR ((vagal OR vagus) NEAR/4 (stimulat*)))) **AND (**TI=(Systematic OR metaanalysis OR meta-analysis) OR AB=(Systematic OR metaanalysis OR meta-analysis OR Cochrane OR Embase OR MEDLINE OR PubMed OR Scopus OR PRISMA**))**
